# Effect of Vapor Pressure During the Steam Coating Treatment on Structure and Corrosion Resistance of the Mg(OH)_2_/Mg-Al LDH Composite Film Formed on Mg Alloy AZ61

**DOI:** 10.3390/ma11091659

**Published:** 2018-09-08

**Authors:** Kae Nakamura, Yuta Shimada, Tomohiro Miyashita, Ai Serizawa, Takahiro Ishizaki

**Affiliations:** 1 Department of Precision Machinery Engineering, College of Science and Technology, Nihon University, 7-24-1 Narashinodai, Funabashi-shi, Chiba 274-8501, Japan; 2 Materials Science and Engineering, Graduate School of Engineering and Science, Shibaura Institute of Technology, 3-7-5 Toyosu, Koto-ku, Tokyo 135-8548, Japan; mb17019@shibaura-it.ac.jp (Y.S.); mb18030@shibaura-it.ac.jp (T.M.); 3 Department of Materials Science and Engineering, College of Engineering, Shibaura Institute of Technology, 3-7-5 Toyosu, Koto-ku, Tokyo 135-8548, Japan; serizawa@sic.shibaura-it.ac.jp

**Keywords:** Mg alloy, steam coating, Mg(OH)_2_, Mg-Al layered double hydroxide, composite film, corrosion resistance

## Abstract

Corrosion resistant films with almost the same film thickness were prepared on the magnesium alloy AZ61 by steam coating at different vapor pressure and treatment times. The effect of the vapor pressure on the structures and the corrosion resistance of the films was investigated by using FE-SEM, SEM-EDX, GAXRD, and potentiodynamic polarization curve measurements in a 3.5 mass percentage NaCl aqueous solution. These studies clarified that the interlayers of Mg-Al Layered Double Hydroxide (LDHs) increased and its structure became non-uniform with an increase in the vapor pressure. The corrosion current density slightly increased with an increase in the vapor pressure during the treatment, but pitting corrosion occurred at both low and high vapor pressures. These results indicate that water molecules were pushed into an interlayer of Mg-Al LDHs by high vapor pressure. Consequently, the interlayer distance of Mg-Al LDH was widened and the cracks were generated in the anti-corrosive film. On the other hand, the Mg-Al LDH with an insufficiently large interlayer distance could not fill the cracks in the Mg(OH)_2_ crystallites and caused pitting corrosion when the vapor pressure was low.

## 1. Introduction

In recent years, although many resins have been widely used as materials for industrial products due to the lightweight property, it is still difficult to manufacture some products made from resins from the viewpoint of performance and cost. Therefore, the demand for metallic materials is still high [1]. Magnesium (Mg) alloys have excellent mechanical and electrical properties [2,3,4], which means they have been applied to some electronic devices. In addition, Mg alloys are expected to be applied to transportation equipment because they are lightweight [5,6,7]. On the other hand, the poor corrosion resistance prevents them from being used practically. Various surface treatment techniques have been developed to overcome this issue. For example, a chemical conversion treatment and an anodic oxidation technique, which are applied to aluminum (Al) alloys [8,9,10,11,12,13,14,15] and steel [16,17,18,19], have also been applied to Mg alloys to impart corrosion resistance [20,21,22,23,24,25,26]. Many conventional surface treatment techniques have been carried out by immersing a metal substrate in a treatment solution. The treatment solution can contain toxic substances [27,28,29]. Thus, it is essential to develop an environmentally-friendly surface treatment method without any toxic substance.

A “steam coating treatment” technique has been developed by our research group and it can prepare anticorrosive film on the metal substrates in the enclosed autoclave [30]. The film formed by this technique is the metal-originating hydroxide and oxide film on the metal substrates by reacting with high-pressure steam at specific temperatures. We have investigated the corrosion resistance of the film prepared on the Mg alloys such as AZ31, AMCa602, and AZCa612 and are formed by steam coating with ultrapure water as a steam source. These studies clarified that the anticorrosive film prepared on the Mg alloys consisted mainly of Mg(OH)_2_ and Mg-Al layered double hydroxide (LDH) [30,31,32]. During the steam coating, Mg(OH)_2_ begins to form at a relatively low temperature such as 80 °C to 100 °C and Mg-Al LDHs, which are formed at a higher temperature of 130 °C to 160 °C [33]. 

In particular, the formation mechanism of Mg-Al LDHs during steam coating was considered to be similar to a solid-phase reaction unlike the solution process because very few ions were likely generated in the steam treatment. In other words, after Mg(OH)_2_ was formed, some Al atoms as a solid solution in the Mg alloy might be incorporated into the Mg(OH)_2_ crystal by diffusion of the Al atoms. Consequently, to compensate for the charge balance, carbonate ions originating from the CO_2_ in the enclosed autoclave could be intercalated as interlayer anions of the Mg-Al LDHs. Therefore, film formation in a steam environment can be performed more efficiently than that in a solution due to the absence of dissolution of metal ions [34,35,36,37]. The steam coating does not require toxic substances and high energy, which is why the process has a low environmental impact.

As previously mentioned, it is highly desirable to prepare the high corrosion resistant film on the metal substrates in a short amount of time even though the steam coating has been carried out at various treatment conditions in order to utilize the process industrially. This may be realized by making vapor pressure higher in the autoclave during the treatment because the water molecules have high kinetic energy due to an increase in the vapor pressure. In addition, the film formation reaction can be accelerated by an increase in the energy. Moreover, it is expected that the change in vapor pressure induces the behavior of the crystal growth for the anticorrosive film, which resulted in the improvement of the corrosion resistance. However, the effect of the vapor pressure on the characteristics of the film prepared on the Mg alloys by steam coating has not been investigated yet. In this study, we report on the effect of vapor pressure in the enclosed autoclave during the steam coating on the characteristics of the anticorrosive film formed on an Mg alloy. In addition, the relationship between the film structures and the corrosion resistance was also discussed.

## 2. Methods and Materials 

**Figure 1:**
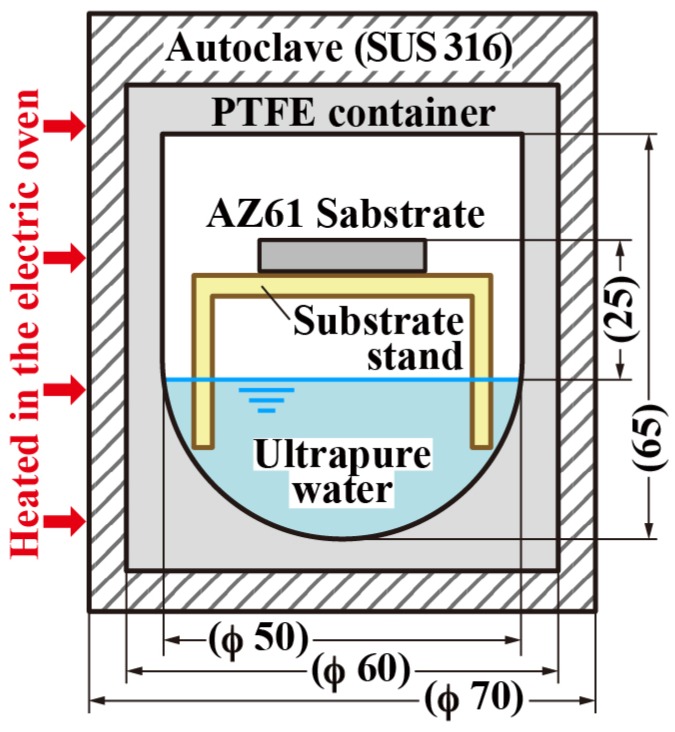
Schematic illustration of experimental set-up for steam coating.

Commercial-rolled magnesium alloy AZ61 (composition: 6.10 mass% Al, 0.64 mass% Zn, 0.28 mass% Mn, 0.009 mass% Si, <0.002 mass% Cu, <0.002 mass% Ni, <0.002 mass% Fe, and the rest is Mg) specimens with a size of 20 mm × 20 mm × 1 mm were used as the substrates. The substrates were mechanically polished using an abrasive paper with a finer grade such as 1200 grit to obtain a flat surface. They were ultrasonically cleaned in absolute ethanol for 10 min and then dried with inert nitrogen gas. The cleaned AZ61 substrates were introduced into a Teflon-lined autoclave with a 100 mL capacity. A total of 20 mL of ultrapure water with a resistance of 18.2 MΩ·cm was placed at the bottom of the autoclave to produce steam. The cleaned AZ61 was placed on the substrate stage made of SUS304 wire mesh and covered with PTFE tape in the autoclave. The distance between the water surface and the substrate stage was ~25 mm. A schematic illustration of the steam-coating process is shown in Figure 1. The autoclave was heated to generate steam and held at a constant pressure for a predetermined time so that the film thickness of all samples could be kept constant (~20 μm). All treatment conditions are listed in Table 1. The values of water vapor pressure were measured during the steam coating in the autoclave by using a pressure sensor (KEYENCE, AP-14S, Osaka, Japan) in the preliminary experiment. The pressure values were ~90% of the saturated vapor pressure at each temperature. The films were prepared at predetermined pressures under identical conditions on three occasions in order to confirm the reproducibility.

The surface morphologies of the films on the AZ61 substrates were investigated by a field-emission scanning electron microscope (FE-SEM, JSM-IT300HR, JEOL, Tokyo, Japan) at 15 keV. The crystallographic structure of the compound constituting the film was examined by glancing at angle X-ray diffraction (GAXRD; Ultima IV, Rigaku, Tokyo, Japan) at a glancing angle of 1° with Cu Kα radiation (40 kV, 40 mA) within the range of 5° to 80° and at a scanning rate of 2θ = 4° min^−1^. The composition of the prepared films was analyzed using energy-dispersive X-ray (FE-SEM-EDX) analysis. 

To study the corrosion resistance, electrochemical measurements were carried out and potentiodynamic polarization curves were recorded. All electrochemical measurements were performed in 3.5 mass% NaCl aqueous solution at room temperature using a computer-controlled potentiostat (Princeton Applied Research, VersaSTAT3, Oak Ridge, TN, USA). The film-coated AZ61 and a Pt mesh were used as the working and counter electrodes, respectively. The reference electrode was Ag/AgCl sat. KCl. Each sample was immersed in the NaCl solution for 30 min, which allowed the system to be stabilized, and potentiodynamic polarization curves were subsequently measured with respect to the open circuit potential (OCP) at a scanning rate of 0.5 mV/s from −100 to +800 mV. The corrosion potential, *E*_corr_, and corrosion current density, *i*_corr_, values were determined from the experimental potentiodynamic curves using the program CorrView to obtain the fitting parameters.

## 3. Results and Discussions

### 3.1. Morphologies of the Anticorrosive Films

**Figure 2:**
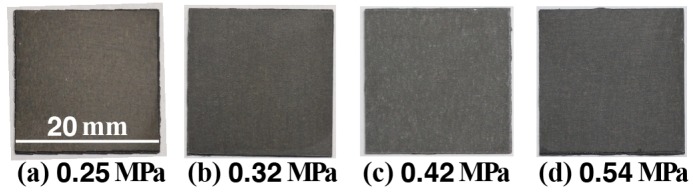
Appearances of the films formed on AZ61 by steam coating under different treatment conditions.

**Figure 3:**
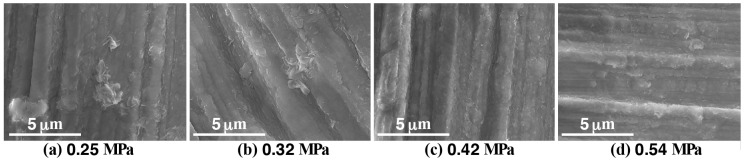
FE-SEM images of the films formed on AZ61 by steam coating under different conditions.

**Figure 4:**
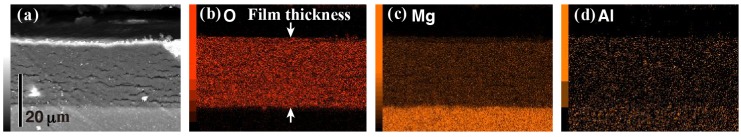
Cross-sectional image (**a**) and elemental mappings of the cross-sections (**b**), (**c**), (**d**) for Mg, Al, and O of the sample (C) treated at 0.42 MPa.

**Figure 5:**
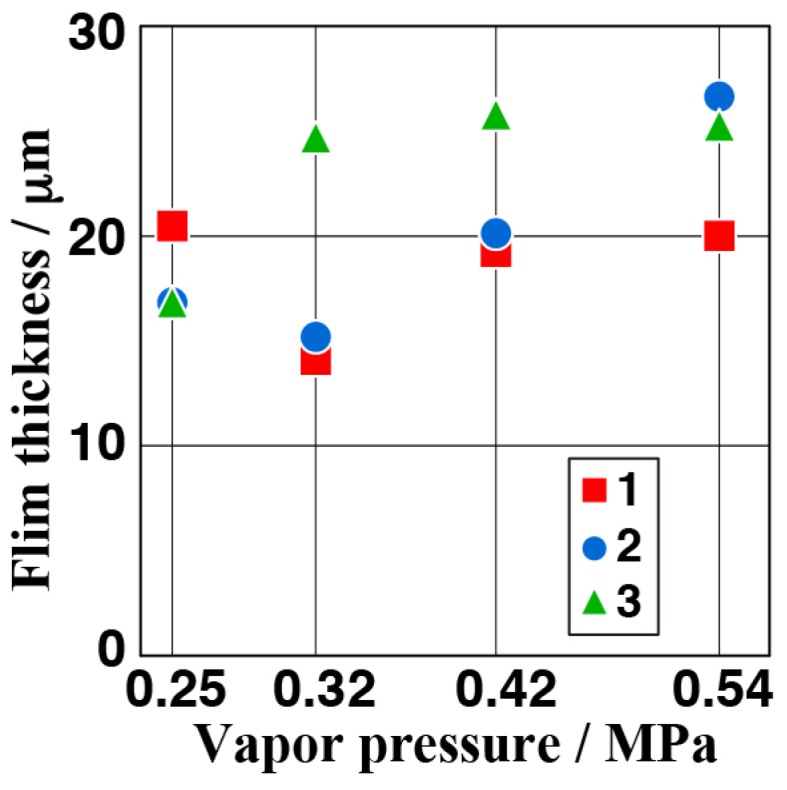
Thickness of anticorrosive film for all samples.

Figure 2 shows the appearance of the samples (A) to (D) after steam coating under treatment conditions listed in Table 1. The appearance of all samples was almost the same and it looked brownish gray independent of the treatment conditions. The appearances of the anticorrosive films formed by steam coating are related to the film thickness. Therefore, the fact that their color was the same indicates that the film thickness was almost the same. Figure 3 shows FE-SEM images of the samples (A) to (D). Small plate-shaped products can be observed on all sample surfaces. In samples (A) and (B), the products were clustered while, in samples (C) and (D), the products looked to be dispersed and were stacked to the surface. The atomic concentrations measured using SEM-EDX in the area of 18.2 μm × 13.7 μm on the anticorrosive film are listed in Table 2. Each atomic concentration for all of the samples was almost the same. In particular, the amount of O almost equaled the sum of twice the amount of Mg and three times the amount of Al, which indicates that Mg(OH)_2_ and the Al compound containing oxygen were formed on the AZ61 independent of vapor pressure values. The Al compound was probably attributed to Mg-Al LDH and Al(OH)_3_ since some researchers reported that amorphous filamentary or colloidal Al(OH)_3_ was formed at the initial formation stage of Mg-Al LDH [38]. Figure 4 shows the cross-sectional SEM and elemental Mg, Al, and O mapping images of the film for the sample (C). Focusing on the mapping image of oxygen, it was uniformly distributed in a band shape. The width of this band was measured as the film thickness. To confirm the reproducibility of the film thickness for a preparation condition, the film was prepared under the same condition three times. The relationship between vapor pressure values in the autoclave and the film thickness obtained from the FE-SEM observation is shown in Figure 5. The average measurement of the film thickness was estimated to be 20.4 ± 6.3 μm. This indicates our steam coating process can control the film thickness by controlling the process condition.

**Figure 6:**
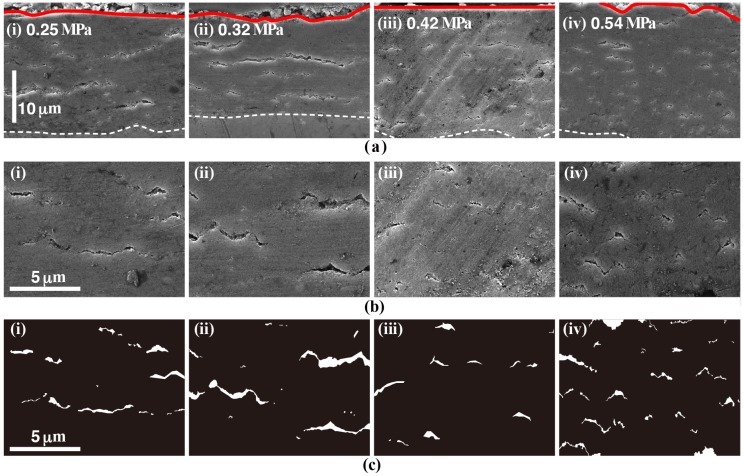
Cross-sectional SEM images (**a**), their enlarged images (**b**), and images with cracks highlighted (**c**) of the film for all samples.

Figure 6 shows (a) cross-sectional SEM images, (b) their enlarged images, and (c) images with cracks highlighted of the film on all samples. In both samples (A) and (B), the development of some long cracks can be observed in the film. The width of the cracks in sample (B) was larger than that of the cracks in sample (A). Although the size of the cracks in the film of samples (C) and (D) was smaller than that of samples (A) and (B), the existence amounts of the cracks increased. These cross sectional FE-SEM images revealed that the ratio of the crack formation could depend on the water vapor pressure during the film formation process. These results indicate that the difference in the water vapor pressure can affect the crystal growth of the film. In addition, water vapor pressure could be one key factor for the formation of the dense film.

### 3.2. Crystal Phases of the Anticorrosive Film

**Figure 7:**
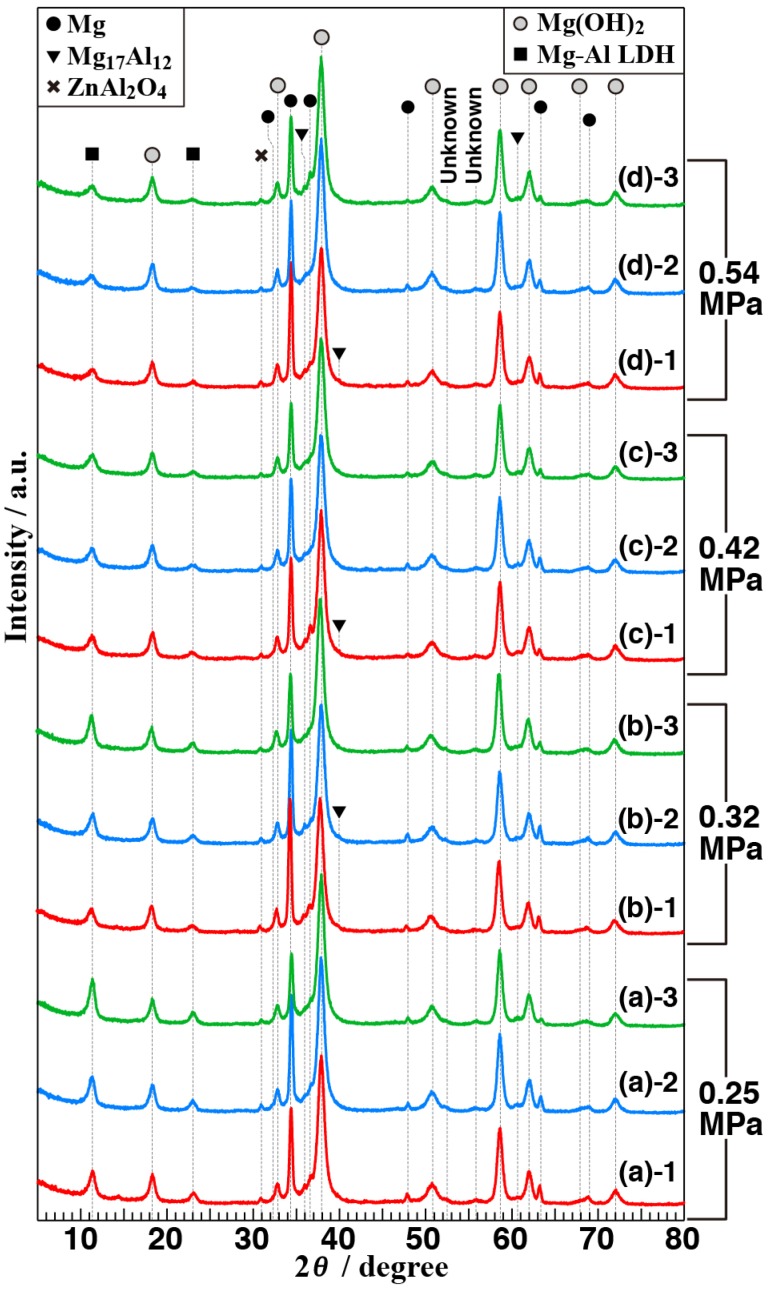
GAXRD patterns of three sets of data per condition of the films formed on AZ61 by steam coating under different conditions: (**a**) 0.25, (**b**) 0.32, (**c**) 0.42, and (**d**) 0.54 MPa.

Figure 7 shows GAXRD patterns of the samples (A) to (D). Each sample was prepared under an identical condition three times, which led to three GAXRD patterns for each sample to be shown in Figure 7. Some peaks attributable to Mg were clearly observed at 2θ = 32.2°, 34.3°, 36.6°, 47.9°, 63.2°, and 69.0° in all GAXRD patterns. In addition, small peaks attributable to Mg_17_Al_12_ and ZnAl_2_O_4_ were also detected in all XRD patterns. Three small peaks at around 2θ = 36.0°, 40.0°, and 61.0° can be assigned to the 411, 332, and 444 diffraction peaks of Mg_17_Al_12_ as an intermetallic compound and the Mg_17_Al_12_ has been commonly found in Mg-Al-Zn system alloys [39,40,41]. A small peak at around 2θ = 31.0° could seem to be attributable to 220 reflections of the spinel type ZnAl_2_O_4_, according to the International Centre for Diffraction Data Powder Diffraction File (ICDD-PDF) number 00-005-0669. Czerwinski reported that the spinel type MgAl_2_O_4_ was detected when the AZ91D Mg alloy was oxidized at a high vapor pressure [42]. Thus, during our steam process, the ZnAl_2_O_4_ may be formed on the AZ61.

A peak at around 2θ = 18° assigned to the 001 reflection of brucite-type Mg(OH)_2_ could be detected from all samples. The diffraction peak of the (011) plane at around 2θ = 38°, which has the highest intensity, according to the ICDD-PDF file number 01-083-0114, can also be observed independent of the vapor pressure. In addition, some peaks observed at around 2θ = 38°, 51°, 59°, 62°, 68°, and 72° in all GAXRD patterns are assigned to the 011, 012, 110, 111, 103, and 201 reflections of Mg(OH)_2_, respectively. These results imply that hexagonal Mg(OH)_2_ are contained in all films. The small peak at around 2θ = 14° observed in the XRD pattern for sample (A-1) which written as (a)-1 in Figure 7 is attributable to the 020 reflection of bohmite-type AlO(OH) (ICDD-PDF no. 01-074-2896). Although this is one of the film components generated by the steam coating, the trace amount can be negligible in this study. In addition to the previously mentioned peaks, two peaks attributable to the hydrotalcite (HT)-like structure are clearly observed, which were assigned to Mg_1–*x*_Al*_x_*(OH)_2_(CO_3_)*_x_*_/2_ · *n*H_2_O (Mg-Al LDH) intercalated with carbonate anions. The two peaks at around 2θ = 11° and 22° were corresponding to the (003) and (006) planes of Mg_1–*x*_Al*_x_*(OH)_2_(CO_3_)*_x_*_/2_·*n*H_2_O, respectively (ICDD-PDF no. 01-089-0461). These results indicate that all films consist of Mg(OH)_2_ and Mg-Al LDH.

**Figure 8:**
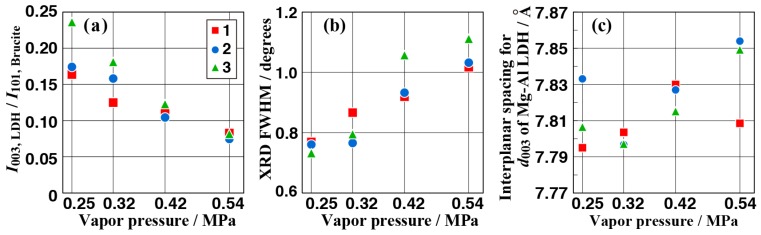
Analysis results based on GAXRD data: the relationship between vapor pressure and the intensity ratio of the 003 reflection peak of Mg-Al LDH to the 110 reflection peak of Mg(OH)_2_ (**a**), XRD FWHM values for intensity peak of the 003 reflection (**b**), and inter-planar spacing for the [003] plane of Mg-Al LDH (**c**).

Figure 8a–c shows the intensity ratio of 003 reflection of Mg-Al LDH (at 2θ = 11.3°) to 011 reflection of Mg(OH)_2_ (at 2θ = 38.0°), full width at half maximum (FWHM) values for intensity peak of 003 reflection of Mg-Al LDH (at 2θ = 11.3°), and inter-planar spacing for [003] plane of Mg-Al LDH calculated from the curve fitting of the peak at around 2θ = 11.3° as a function of vapor pressures, respectively. The ratio in Figure 8a indicates the roughly estimated Mg-Al LDH content in the film. Therefore, when the peak intensity for 003 reflection of Mg-Al LDH became smaller and broader, Mg-Al LDH content could be reduced relatively by increasing the vapor pressure. Moreover, the inter-planar spacing for the [003] plane of Mg-Al LDH tended to increase with the water vapor pressure, which is shown in Figure 8c. These results show that the water vapor pressure during the steam process can affect the crystal growth of the film even though the details are discussed in Section 3.4.

### 3.3. Corrosion Resistance of the Films

**Figure 9:**
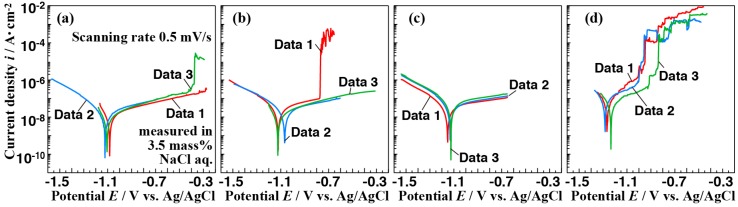
Potentiodynamic polarization curves of the anticorrosive film coated on the AZ61 substrate under different treatment conditions: (**a**) 0.25, (**b**) 0.32, (**c**) 0.42, and (**d**) 0.54 MPa in 3.5 mass% NaCl aqueous solution.

The corrosion resistance of all films prepared on the AZ61 was investigated by potentiodynamic polarization curve measurements. The lower the current density was, the better the corrosion resistance was. Figure 9 shows the potentiodynamic polarization curves of the samples (A) to (D). Each sample was prepared three times under identical conditions. Hence, three potentiodynamic polarization curves for each sample are shown in Figure 9. In Figure 9, the abrupt increase in the current density can be clearly observed in the polarization curves at the potentials of approximately −0.4, −0.8, and −0.8 to −1.0 V for samples (A-3), (B-1), and (D-1, D-2, D-3), respectively. This increase in the current density could be related to the pitting corrosion (Figure S1). The reason of this inference is as follows. In the potentiodynamic polarization measurement, the current density increases when the more electrolyte touches to the metal substrate, which is the work electrode. The abrupt increase in the current density indicates the electrolyte permeated through the pits and cracks in the film and, then, it reached the AZ61 substrate [43]. In contrast, the polarization curves for the samples (C-1, C-2, C-3) indicated a clear passive behavior at more positive potentials than *E*_corr_. The presence of a passive region suggests that the film coated on the AZ61 exhibits highly protective properties in a solution containing Cl^−^ ions. These results indicate that the difference in the vapor pressure during the steam coating process can influence the crystal growth and density of the film. It should be noted that the films prepared at a vapor pressure of 0.54 MPa showed the occurrence of the pitting corrosion while no pitting corrosion was observed for the films prepared at a vapor pressure of 0.42 MPa. These results reveal that vapor pressures higher than approximately 0.54 MPa are not suitable for preparing highly corrosive resistant films on AZ61.

### 3.4. Effect of Vapor Pressure on the Structure and the Corrosion Resistance of the Film

**Figure 10:**
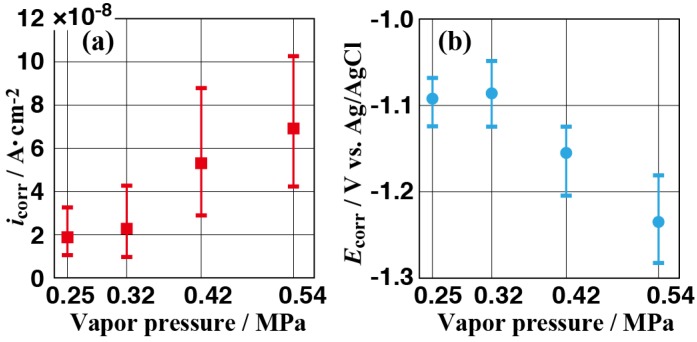
Corrosion current densities (**a**) and corrosion potentials (**b**) determined from the experimental potentiodynamic curves for all samples.

Corrosion current densities (*i*_corr_) and corrosion potentials (*E*_corr_) for the samples prepared at various conditions as a function of vapor pressures are shown in Figure 10a,b, respectively. In Figure 10, the average values are shown as plots and the error bars show the minimum and maximum of all *i*_coor_ and *E*_corr_ values. Their values were obtained from the results of seven repeat polarization curve measurements. The *E*_corr_ tended to shift to a negative direction and the *i*_corr_ values slightly increased with an increase in the vapor pressure. These results indicate that the corrosion resistance of the film tended to improve with a decrease in the water vapor pressure. On the other hand, in terms of the suppression for the occurrence of the pitting corrosion, the film prepared at 0.42 MPa showed the best behavior within the preparation conditions investigated. The difference in the corrosion resistance of the film can be related to the film structure. With respect to the film structure, the produced amount of Mg-Al LDH decreased as the vapor pressure increased. At the same time, the interlayer distance of Mg-Al LDH tended to expand. Millange et al. investigated the structural changes of the Mg-Al LDH in terms of the desorption of the carbonate ions in the Mg-Al LDH during the calcination process using high-temperature XRD (HTXRD) [44]. They clarified that the dehydration process induced a decrease in the basal spacing of Mg-Al LDH when the sample was calcined at 240 °C. In contrast, in this study, the interlayers of Mg-Al LDH were expanded with an increase in the water vapor pressure. This might be due to an increase in the entrapment amount of water vapor in the interlayers. This expansion of the interlayer in the Mg-Al LDH may induce a change in the film structure such as a change in the film denseness. The Mg-Al LDH content in the film were roughly estimated using the intensity ratio of 003 reflection of Mg-Al LDH (at 2θ = 11.3°) to 011 reflection of Mg(OH)_2_ (at 2θ = 38.0°), which is shown in Figure 8a. Based on Figure 8a and Figure 9, when the intensity ratio of the 003 reflection of Mg-Al LDH to the 011 reflection of Mg(OH)_2_ was in the range of 0.10 to 0.18, the pitting corrosion was barely observed. On the other hand, when the intensity ratio of the 003 reflection of Mg-Al LDH to the 011 reflection of Mg(OH)_2_ was outside the range of 0.10 to 0.18, the pitting corrosion could occur. In particular, when the ratio of Mg-Al LDH to Mg(OH)_2_ was more than 0.18, the corrosion resistance of the film was considerably lower. These results indicate that the existent ratio of Mg-Al LDH to Mg(OH)_2_ would be an important key factor for preparing a superior corrosion resistant film. In general, the denseness is an important factor for improving the protect performance of the film. Therefore, the existent ratio of Mg-Al LDH to Mg(OH)_2_ would be an important factor for preparing superior corrosion resistant film by a steam coating process.

## 4. Conclusions 

Corrosion-resistant films with almost the same film thickness were prepared on the magnesium alloy AZ61 by steam coating at different vapor pressures. All films prepared on AZ61 were characterized by FE-SEM, SEM-EDX, GAXRD, and potentiodynamic polarization curve measurements in a 3.5 mass% NaCl aqueous solution. XRD studies revealed that the film was composed mainly of Mg(OH)_2_ and carbonate-based Mg-Al LDHs and the produced amount of Mg-Al LDHs decreased with increasing vapor pressure in the enclosed autoclave. In addition, detailed analysis of the peak positions of the 003 reflections for Mg-Al LDH of GAXRD patterns revealed that the interlayer distance of the Mg-Al LDH tends to increase as the vapor pressure in the autoclave increased. 

A comparison of the results of all analyses revealed the effect of treatment conditions on the structure and corrosion resistance of the film as follows. As the produced amount of Mg-Al LDH increased, the corrosion current density tended to decrease slightly. However, this has little effect on the improvement of the corrosion resistance. When the vapor pressure was high, there were many small cracks in the film and pitting corrosion occurred frequently. The difference in the vapor pressure during the steam coating process can influence the crystal growth and denseness of the film. The results of the XRD and electrochemical measurements revealed that, when the intensity ratio of the 003 reflection of Mg-Al LDH to the 011 reflection of Mg(OH)_2_ was in the range of 0.10 to 0.18, pitting corrosion can be hardly observed. However, pitting corrosion could occur when the intensity ratio was outside this range. In particular, when this ratio was more than 0.18, the corrosion resistance of the film was considerably lowered. These results indicate that the existent ratio of Mg-Al LDH to Mg(OH)_2_ is an important key factor in preparing superior corrosion-resistant films. All results showed that vapor pressures higher than approximately 0.54 MPa were not suitable for preparing highly corrosion resistant films on AZ61.

With a growing demand for Mg alloys and an increasing focus on environmental issues, our steam coating treatment can provide an effective means of improving the corrosion performance of large, complex-shaped Mg alloy components. The influences of the steam-coating treatment conditions on the structures and corrosion resistance of the film prepared on AZ61 is expected to be useful for applying steam coating to various industrial fields using Mg alloys.

## Figures and Tables

**Table 1:** Treatment conditions for steam coating of all samples on AZ61.

Sample	Vapor Pressure (MPa)	Temperature (°C)	Treatment Time (h:min)	Target Value of Film Thickness (m)
(A) (B) (C) (D)	0.25, (0.27)* 0.32, (0.36)* 0.42, (0.48)* 0.54, (0.62)*	130 140 150 160	9:30 5:30 4:15 3:20	20.0

* The values show saturated vapor pressure at each temperature.

**Table 2:** Element concentrations of the anticorrosive film detected by EDX.

Sample	at. %				
	C	O	Mg	Al	Zn
(A) (B) (C) (D)	4.22 6.60 4.80 5.26	65.55 63.19 64.75 64.47	28.50 28.29 27.75 27.97	1.68 1.82 2.68 2.25	0.05 0.10 0.02 0.04

## Supplementary Materials

The following are available online at http://www.mdpi.com/1996-1944/11/9/1659/s1, Figure S1: Appearances of the films formed on AZ61 by steam coating under different treatment conditions: (a) 0.25, (b) 0.32, (c) 0.42, and (d) 0.54 MPa after the electrochemical polarization measurement.

## References

[1] (2018). United States Geological Survey, Bureau of Mines Minerals Yearbook. http://minerals.usgs.gov/minerals/pubs/usbmmyb.html.

[2] Nakatsugawa I., Umehara H., Akimoto M., Shimamura K., Ono S., Takaya M., Tsubota T., Editorial Committee of Handbook of Advanced Magnesium Technology (2000). Corrosion and surface treatment of magnesium alloy. Handbook of Advanced Magnesium Technology (in Japanese).

[3] Mordike B.L., Ebert T. (2001). Magnesium properties—Applications—Potential. Mater. Sci. Eng. A.

[4] Wang X.J., Xu D.K., Wu R.Z., Chen X.B., Peng Q.M., Jin L., Xin Y.C., Zhang Z.Q., Liu Y., Chen X.H. (2018). What is going on in magnesium alloys?. J. Mater. Sci. Technol..

[5] Kumar D.S., Sasanka C.T., Ravindra K., Suman K.N.S. (2015). Magnesium and its alloys in automotive applications—A review. Am. J. Mater. Sci. Technol..

[6] Pollock T.M. (2010). Weight loss with magnesium alloys. Science.

[7] Aghion E., Bronfin B., Eliezer D. (2001). The role of the magnesium industry in protecting the environment. J. Mater. Process. Technol..

[8] Metroke T.L., Kachurina O., Knobbe E.T. (2002). Spectroscopic and corrosion resistance characterization of GLYMO–TEOS Ormosil coatings for aluminum Aaloy corrosion inhibition. Prog. Org. Coat..

[9] Takai M., Takaya M. (2008). Influence of conversion coating on magnesium and aluminum alloys by adhesion method. Mater. Trans..

[10] Sakuragi N., Yamamoto S., Koide Y. (2007). A self-assembled monolayers assisted solid-state conversion of boehmite particles to aluminum oxide film. J. Am. Chem. Soc..

[11] Kulinich S.A., Farzaneh M., Du X.W. (2007). Growth of corrosion-resistant manganese oxide coatings on an aluminum alloy. Inorg. Mater..

[12] He T., Wang Y.C., Zhang Y.J., Lv Q., Xu T., Liu T. (2009). Super-hydrophobic surface treatment as corrosion protection for aluminum in seawater. Corros. Sci..

[13] Konieczny J., Dobrzański L.A., Labisz K., Duszczyk J. (2004). The influence of cast method and anodizing parameters on structure and layer thickness of aluminium alloys. J. Mater. Process. Technol..

[14] Saenz de Miera M., Curioni M., Skeldon P., Thompson G.E. (2008). Modelling the anodizing behavior of aluminium alloys in sulphuric acid through alloy analogues. Corros. Sci..

[15] Twite R.L., Bierwagen G.P. (1998). Review of alternatives to chromate for corrosion protection of aluminum aerospace alloys. Prog. Org. Coat..

[16] Tallman D.E., Spinks G., Dominis A., Wallace G.G. (2002). Electroactive conducting polymers for corrosion control. J. Solid State Electr..

[17] Kure K., Konno Y., Tsuji E., Skeldon P., Thompson G.E., Habazaki H. (2012). Formation of self-organized nanoporous anodic films on Type 304 stainless steel. Electrochem. Commun..

[18] Song Y.K., Mansfeld F. (2006). Development of a molybdate–phosphate–silane–silicate (MPSS) coating process for electrogalvanized steel. Corros. Sci..

[19] Hara M., Ichino R., Okido M., Wada N. (2003). Corrosion protection property of colloidal silicate film on galvanized steel. Surf. Coat. Technol..

[20] Li O.L., Tsunakawa M., Shimada Y., Nakamura K., Nishinaka K., Ishizaki T. (2017). Corrosion resistance of composite oxide film prepared on Ca-added flame-resistant magnesium alloy AZCa612 by micro-arc oxidation. Corros. Sci..

[21] Chong K.Z., Shih T.S. (2003). Conversion-coating treatment for magnesium alloys by a permanganate-phosphate solution. Mater. Chem. Phys..

[22] Sharma A.K., Rani R.U., Mayanna S.M. (2001). Thermal studies on electrodeposited black oxide coating on magnesium alloys. Thermochim. Acta.

[23] Montemora M.F., Ferreira M.G.S. (2007). Electrochemical study of modified bis-[triethoxysilylpropyl] tetrasulfide silane films applied on the AZ31 Mg alloy. Electrochim. Acta.

[24] Blawert C., Dietzel W., Ghali E., Song G. (2006). Anodizing treatments for magnesium alloys and their effect on corrosion resistance in various environments. Adv. Eng. Mater..

[25] Zeng R.C., Cui L.Y., Jiang K., Liu R., Zhao B.D., Zheng Y.F. (2016). In vitro corrosion and cytocompatibility of a microarc oxidation coating and poly(L-lactic acid) composite coating on Mg−1Li−1Ca alloy for orthopedic implants. Appl. Mater. Interfaces.

[26] Duan G.Q., Yang L.X., Liao S.G., Zhang C.Y., Lu X.P., Yang Y., Zhang B., Wei Y., Zhang T., Yu B.X. (2018). Designing for the chemical conversion coating with high corrosion resistance and low electrical contact resistance on AZ91D magnesium alloy. Corros. Sci..

[27] Kendig M., Jeanjaquet S., Addison R., Waldrop J. (2001). Role of hexavalent chromium in the inhibition of corrosion of aluminum alloys. Surf. Coat. Technol..

[28] Meng Q., Frankel G.S. (2004). Effect of copper content on chromate conversion coating protection of 7xxx-T6 alminum alloys. Corrosion.

[29] Rocco A.M., Nogueira T.M.C., Simao R.A., Lima W.C. (2004). Evaluation of chromate passivation and chromate conversion coating on 55% Al–Zn coated steel. Surf. Coat. Technol..

[30] Ishizaki T., Chiba S., Watanabe K., Suzuki H. (2013). Corrosion resistance of Mg-Al layered double hydroxide container-containing magnesium hydroxide films formed directly on magnesium alloy by chemical-free steam coating. J. Mater. Chem. A.

[31] Kamiyama N., Panomsuwan G., Yamamoto E., Sudare T., Saito N., Ishizaki T. (2016). Effect of treatment time in the Mg(OH)_2_/Mg-Al LDH composite film formed on Mg alloy AZ31 by steam coating on the corrosion resistance. Surf. Coat. Technol..

[32] Ishizaki T., Kamiyama N., Watanabe K., Serizawa A. (2015). Corrosion resistance of Mg(OH)_2_/Mg–Al layered double hydroxide composite film formed directly on combustion-resistant magnesium alloy AMCa602 by steam coating. Corros. Sci..

[33] Nakamura K., Tsunakawa M., Shimada Y., Serizawa A., Ishizaki T. (2016). Formation mechanism of Mg-Al layered double hydroxide-containing magnesium hydroxide films prepared on Ca-added flame-resistant magnesium ally by steam coating. Surf. Coat. Technol..

[34] Ke C., Wu Y., Qiu Y., Duan J.H., Birbilis N., Chen X.B. (2016). Influence of surface chemistry on the formation of crystalline hydroxide coatings on Mg alloys in liquid water and steam systems. Corros. Sci..

[35] Kozawa T., Yanagisawa K., Yoshida A., Onda A., Suzuki Y. (2013). Preparation of β-CaSiO_3_ powder by water vapor-assisted solid-state reaction. J. Ceram. Soc. Jpn..

[36] Kozawa T., Yanagisawa K., Yoshida A., Suzuki Y. (2013). Water vapor-assisted solid-state reaction for the synthesis of nanocrystalline BaZrO_3_ powder. J. Ceram. Soc. Jpn..

[37] Din R.U., Jellesen M.S., Ambat R. (2015). Performance comparison of steam-based and chromate conversion coatings on aluminum alloy 6060. Corrosion.

[38] Zhang G., Wu L., Tang A., Chen X.B., Ma Y.L., Long Y., Peng P., Ding X.X., Pan H.L., Pan F.S. (2018). Growth behavior of MgAl-layered double hydroxide films by conversion of anodic films on magnesium alloy AZ31 and their corrosion protection. Appl. Surf. Sci..

[39] Hort N., Huang Y., Kainer K.U. (2006). Intermetallics in magnesium alloys. Adv. Eng. Mater..

[40] Feliu Jr. S., Maffiotte C., Samaniego A., Galván J.C., Barranco V. (2011). Effect of the chemistry and structure of the native oxide surface film on the corrosion properties of commercial AZ31 and AZ61 alloys. Appl. Surf. Sci..

[41] El-Morsy A., Ismail A., Waly M. (2008). Microstructural and mechanical properties evolution of magnesium AZ61 alloy processed through a combination of extrusion and thermomechanical processes. Mater. Sci. Eng. A.

[42] Czerwinski F. (2002). The oxidation behaviour of an AZ91D magnesium alloy at high temperatures. Acta Mater..

[43] Kannan M.B., Raja V.S. (2007). Influence of Heat treatment and scandium addition on the electrochemical polarization behavior of Al-Zn-Mg-Cu-Zr alloy. Metall. Mater. Trans. A.

[44] Millange F., Walton R.I., O’Hare D. (2000). Time-resolved in situ X-ray diffraction study of the liquid-phase reconstruction of Mg-Al-carbonate hydrotalcite-like compounds. J. Mater. Chem..

