# Supplementary material for: Effect of Vapor Pressure During the Steam Coating Treatment on Structure and Corrosion Resistance of the Mg(OH)2/Mg-Al LDH Composite Film Formed on Mg Alloy AZ61
Source: Materials (Basel). 2018 Sep 8;11(9):1659. doi: 10.3390/ma11091659 (PMC6164506; doi:10.3390/ma11091659)
Supplement: Supplementary file 1 [file materials-11-01659-s001.pdf]

Supplementary Materials

Effect of Vapor Pressure During the Steam Coating Treatment on Structure and Corrosion Resistance of the $Mg(OH)_2$/Mg-Al LDH Composite Film Formed on Mg Alloy AZ61

Kae Nakamura, Yuta Shimada, Tomohiro Miyashita, Ai Serizawa and Takahiro Ishizaki

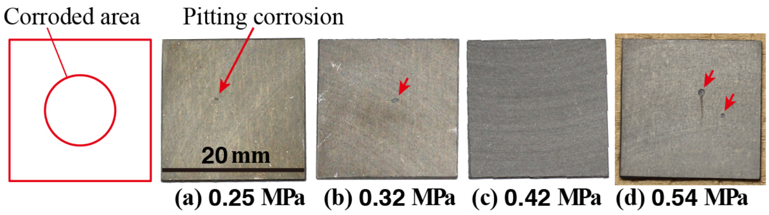

Figure S1. Appearances of the films formed on AZ61 by steam coating under different treatment conditions: (a) 0.25, (b) 0.32, (c) 0.42, and (d) 0.54 MPa after the electrochemical polarization measurement.
